# Expression of Antimicrobial Peptide (AMP), Cecropin B, in a Fused Form to SUMO Tag With or Without Three-Glycine Linker in *Escherichia coli* and Evaluation of Bacteriolytic Activity of the Purified AMP

**DOI:** 10.1007/s12602-021-09797-1

**Published:** 2021-05-20

**Authors:** A Rom Park, Seon Woong Kim, Soon Young Kim, Kwang-Chul Kwon

**Affiliations:** 1 Department of Biological Sciences, Andong National University, Andong, Korea; 2 MicroSynbiotiX Ltd, 11011 N Torrey Pines Rd Ste #135, La Jolla, CA 92037 USA

**Keywords:** Cecropin B, Antimicrobial peptide, Small ubiquitin-related modifier (SUMO), Codon optimization, *Escherichia coli*, Chloroplast

## Abstract

Current antibiotics have limited action mode, which makes it difficult for the antibiotics dealing with the emergence of bacteria resisting the existing antibiotics. As a need for new bacteriolytic agents alternative to the antibiotics, AMPs have long been considered substitutes for the antibiotics. Cecropin B was expressed in a fusion form to six-histidine and SUMO tags in *Escherichia coli.* Six-histidine tag attached to SUMO was for purification of SUMO-cecropin B fusion proteins and removal of the SUMO tag from cecropin B. Chimeric gene was constructed into pKSEC1 vector that was designed to be functional in both *Escherichia coli* and chloroplast. To maximize translation of the fusion protein, sequences were codon-optimized. Four different constructs were tested for the level of expression and solubility, and the construct with a linker, 6xHisSUMO3xGly-cecropin B, showed the highest expression. In addition, cleavage of the SUMO tag by SUMOase in the three fusion constructs which have no linker sequence (3xGly, three glycines) was not as efficient as the construct with the linker between SUMO and cecropin B. The cleaved cecropin B showed bacteriolytic activity against *Bacillus subtilis* at a concentration of 0.0625 μg/μL, while cecropin B fused to SUMO had no activity at a higher concentration, 0.125 μg/μL. As an expression system for AMPs in prokaryotic hosts, the use of tag proteins and appropriate codon-optimization strategy can be employed and further genetic modification of the fusion construct should help the complete removal of the tag proteins from the AMP in the final step of purification.

**Supplementary Information:**

The online version contains supplementary material available at 10.1007/s12602-021-09797-1.

## Introduction

A dire report shows that over one million people die due to antibiotic-resistance bacteria every year [1], which is largely attributed to the overuse of antibiotics for the past several decades. Despite a continuous demand for new antibiotics, the discovery of them has remained deadlock. The growing difficulty of developing new antibiotics has put AMPs under attention.

AMPs are found in various organisms from prokaryote to human and have a wide range of anti-bacterial activity [2]. AMPs are generally defined as a small peptide group ranging from 10 to 50 amino acids in size with positive net charge ranging from +2 to +11 [3, 4]. Interaction of positively charged AMPs with negatively charged bacterial membrane triggers cracks in the membrane, resulting in forming pores and eventual death of bacteria [4]. In addition to the pore-forming AMPs, some AMPs can translocate bacterial membrane barrier by self-promoted uptake and target key cellular processes such as macromolecule synthesis (DNA, RNA, protein, and cell wall), protein folding, and enzyme activity [5–12]. This multi-hit action mechanism can not only increase the efficacy of AMPs, but also help evade resistance development [4, 5, 8, 13, 14]. Further, neutral net charge of mammalian membrane attributed by the abundance of zwitterionic phospholipids can protect mammalian cells from attack by AMPs. The hydrophobic interaction between the mammalian cell membrane and AMPs is much weaker than the electrostatic interaction between the bacterial membrane and AMPs so the conformational change of mammalian cell membranes is not feasible by AMPs [4, 5, 15, 16]. Moreover, the mammalian cell membranes are stabilized by the embedded cholesterols; hence, the activity of AMPs in mammalian cell membranes becomes suppressed [15]. In addition to inherent anti-infective, AMPs also show a wide range of immunomodulatory activities, which helps clearance of bacteria from the host [11].

Considering the small size of peptide, the chemical synthesis appears an easy and straightforward method for the production of AMPs. However, chemical synthesis is not cost-effective for industrial-level production and the synthesis of the peptides longer than 50 amino acids is not suitable [17]. Use of sizable bioreactors of bacteria or yeast can address the issues mentioned above because those biological systems do not require expensive active pharmaceutical ingredients (API) and need to use toxic chemical solvents [18–24]. However, some issues still remain challenging such as possible toxic effects of expressed AMPs to the hosts, low yield caused by instability of AMPs, difficulty of purification, and isolation of authentic AMPs [25].

To address the issues, we have already developed a new expression vector system, pKSEC1, equipped with SUMO and 6xHis tag systems [26]. The attached tags can increase solubility of AMPs by increasing overall hydrophilicity, stability of AMPs by preventing them from protein degradation, protect host cells from possible toxic effect of the AMPs by reducing net positive charge, and make the purification/isolation of AMPs straightforward by providing affinity tag [20, 25, 27–29]. Further, the expression vector can be operable in both *Escherichia coli* and plant chloroplasts since its transcription/translation elements are all derived from prokaryotic origins (bacteria or chloroplasts) so the vector can be immediately used to create transplastomic plants (chloroplast transformants) for large-scale biomass increase when demanded [30, 31]. The plant chloroplast expression platform is free from the risk of endotoxic contamination and robust in the expression of transgenes due to the high copy number of chloroplast genome, up to 10,000 copy numbers per a plant cell [31, 32]. There is almost no positional effect on the transgene expression. This plant platform can also serve as an oral delivery vehicle of biopharmaceuticals expressed in the plant cells [32, 33].

For clinical use, therapeutic proteins/peptides should be authentic amino acid sequences; hence, any tag systems used in the course of expression and purification steps should be removed [27]. Another benefit from using SUMO lies in the fact that SUMO tag can be recognized and cleaved off from AMPs by SUMOase without leaving any unwanted amino acids to AMPs, which is possible because SUMOase recognizes the tertiary structure of SUMO [27, 28, 34]. This characteristic feature makes SUMO/SUMOase system distinct from other proteases including factor Xa, enterokinase, thrombin, and tobacco etch virus protease [35]. In contrast to SUMOase, the proteases recognize linear sequence amino acids so the recognition sometimes can be hindered by steric hindrance or cause erroneous off-target cleavages within target proteins [27], leading to dramatic reduction of final yield of authentic AMPs.

Cecropins were first discovered in Cecropia moth (*Hyalophora cecropia*) pupae [36–38]. The cationic low molecular weight hemolymph proteins appear upon the intrusion of bacteria. Most cecropins are composed of an amphipathic N-terminal portion and a hydrophobic C-terminal portion, and structured into a helix-hinge-helix form [39]. Cecropin B is one of the most extensively studied antibacterial proteins in cecropins. In addition, cecropin-like substances are widely found across lepidopteran, dipteran, and coleopteran insects [40]. Among various cecropins, A, B, and D are the three major cecropins. In the light of antibacterial activity, B shows the highest potency against bacteria so the order follows: B > A ≫ D [36]. Cecropin B is characterized with a molecular weight of 3.84 kDa (35 amino acids) holding +7 net positive charge at pH 7. Out of 35 amino acids, 17 amino acids show hydrophobicity (PepCalc.com).

In this study, we chose cecropin B as a reference AMP to evaluate the relevance of our expression platform. Moreover, we want to describe how this system can be further improved in order to be used as an expression platform for the large-scale production of therapeutic proteins/peptides.

## Materials and Methods

### Cloning of 6xHisSUMO-cecropin B into pKSEC1

All the sequence information for the recombinant protein and peptide are referred to deposited sequences in the National Center for Biotechnology Information (NCBI). The GenBank accession numbers for the sequences of SUMO and cecropin B are NM_003352.4 and M34924.1, respectively. Codon optimizations were carried out as described in a previously published report [31] and the codon-optimized nucleotide sequences were synthesized by Macrogen (Seoul, Republic of Korea) and cloned into pKSEC1 expression vector [26] using *Xba*I and *Nde*I. All the detailed sequences are provided in Supplementary Information Fig. S1. In the naming of constructs, 6xHisSUMO(3xGly)-cecropin B represents all four different constructs of SUMO-cecropin B: 6xHisSUMO (native)-cecropin B (native), 6xHisSUMO (codonoptimized)-cecropin B (native), 6xHisSUMO (codon-optimzied)-cecropin B (codon-optimized), 6xHisSUMO3xGly (codon-optimized)-cecropin B (codon-optimized).

**Fig. 1:**
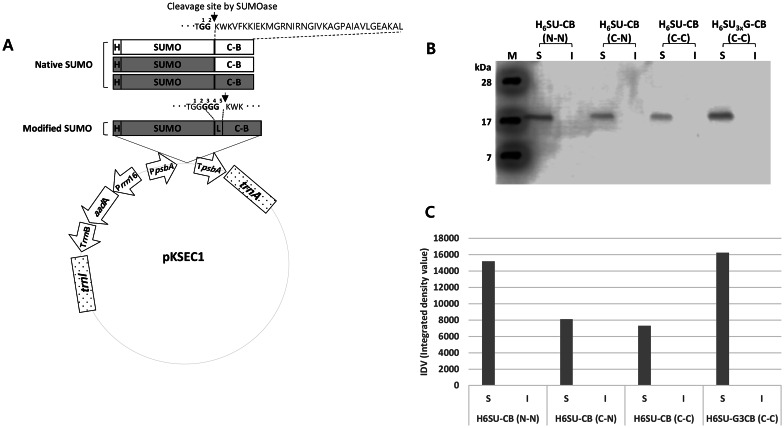
Construction of expression vector. **a** Schematic diagram of expression vector, pKSEC1, with four different 6xHisSUMO-cecropin B chimeric gene constructs. P*rrn*16, 16S rRNA promoter; *aadA*, aminoglycoside 3′ adenylyltransferase gene; T*rrn*B, 3′ UTR of *rrn*B gene; P*psbA, psbA* promoter and 5′ UTR; T*psb*A, 3′ UTR of the *psb*A gene; *trn*I isoleucyl-tRNA; *trn*A alanyl-tRNA. Four constructs of 6xHisSUMO-cecropin B chimeric gene with different codon-optimization strategy are represented by rectangles: white background color means native sequence, and gray background color means codon-optimized sequence. Modified SUMO means the addition of a linker sequence (3 glycines) in between SUMO and cecropin B. Four constructs were driven under the control of *psbA* promoter/5' UTR. H, 6xHistidine; SUMO, Homo sapiens small ubiquitin like modifier 1 (SUMO1); L, 3xGlycine linker; C-B, cecropin B. **b** Comparison of level of expression between four different constructs of 6xHisSUMO-cecropin B in *Escherichia coli* using western blot. H_6_SU-CB, 6xHisSUMO-cecropin B; H_6_SU3xG-CB, 6xHisSUMO3xGly-cecropin B; N, native sequence; C, codon-optimized sequence; S, soluble fraction; I, insoluble fraction; M, protein molecular size marker. The fusion proteins were immunoprobed using anti-histidine antibody. Each lane was loaded with 20 μg of protein. **c** Comparison of band intensities detected in **b**. The band intensities were extrapolated using ImageJ software

### Expression, Quantification, and Immunoblot Assay of SUMO-cecropin B fusion constructs in *Escherichia coli*

The four different constructs of SUMO-crecopin B fusion protein (Fig. 1A) were transformed into *Escherichia coli* (BL21), and the transformants were grown in Terrific Broth (Sigma-Aldrich, St. Louis, MO, USA) containing ampicillin and spectinomycin at a concentration of 100 μg/mL and 50 μg/mL, respectively. The growth was performed in a two-phase way. The culture grown at 37 °C for 3 h at the speed of 200 rpm was further grown at 18 °C overnight. After centrifugation (5800 g, 4 °C, 3 m), the collected cells were resuspended in a ratio of culture cell to buffer (1 × PBS, 1 mg/mL lysozyme, and 1 mM PMSF), 33 to 1 mL. To release expressed proteins, the resuspend cells were ruptured by sonication with a cycle of 5 s on and 5 s off (SONICS VC505, Newtown, CT, USA) for 2 m (80% amplitude), and then proteins released by sonication were separated into soluble and insoluble fractions using centrifugation (9000 g, 4 °C, 20 m). Quantifications of total proteins were done using Bradford (Sigma-Aldrich) assay. Samples having equal amounts of proteins were pre-treated by mixing with 2 × Laemmli Sample Buffer (Bio-Rad, Hercules, CA, USA), and the protein samples were heated at 95 °C for 5 m before entering SDS-PAGE gels. Proteins separated in SDS-PAGE gels were blotted onto PVDF membranes, which were then blocked with 5% skim milk in 1X TBS-T (0.1% Tween 20) for 1 h at room temperature. The blocked membranes were immunoprobed using anti-His antibody (Santa Cruz Biotechnology, Dallas, TX, USA), diluted 1:1000 in the blocking solution, and then incubated at 4 °C for 16 h. The immunoprobed membranes with the primary antibodies were washed with 1X TBS-T buffer for 5 m three times. To detect target protein bands, secondary antibody (goat anti-rabbit IgG-HRP, Santa Cruz), diluted 1:5000 in the blocking solution was incubated at room temperature for 1 h. After washing membranes, ECL buffer was sprayed onto the membranes to develop target protein bands using C-DiGit Blot Scanner (Li-Cor, Lincoln, NE, USA).

### Purification of the Recombinant Cecropin B Using Ni^2+^ Affinity Column

Culture of *Escherichia coli* (BL21) transformants with 6xHisSUMO(3xGly)-cecropin B:pKSEC1 and rupture of the cells were performed as described above. Collected soluble fractions after sonication were filtered through filter paper (Advantec, Tokyo, Japan) and then subject to 0.45-μm syringe filter (Minisart syringe filter, Sartorius Stedim Biotech, Göttingen, Germany). To facilitate the affinity interaction between 6xHis and Ni^2+^ resins (His 60 Ni Suferflow resin, Takara Bio, CA, USA), the mixture of filtrate with resins was incubated inverting slowly for 1 h at 4 °C. After the incubation, Ni^2+^ resin columns were washed with 10 column volume of the equilibration buffer (50 mM sodium phosphate, 300 mM sodium chloride, 10 mM Imidazole; pH 7.4) and 10 column volume of the wash buffer (50 mM sodium phosphate, 300 mM sodium chloride, 40 mM Imidazole; pH 7.4). To elute 6xHisSUMO(3xGly)-cecropin B fusion proteins bound to Ni resins, 10 column volume of elution buffer (50 mM sodium phosphate, 300 mM sodium chloride, 300 mM Imidazole; pH 7.4) was used. Recombinant cecropin B peptides were then isolated from 6xHisSUMO by treatment of SUMOase (Enzynomics, Daejeon, Republic of Korea) at 30 °C for 6 h. To confirm the release of cecropin B peptides from 6xHisSUMO, SDS-PAGE was carried out using NuPAGE™ 4–12% Bis–Tris Gel (Invitrogen, CA, USA).

### Antimicrobial Activity of Cecropin B

To test bacteriolytic activity of the cecropin B peptides, agar diffusion assay was performed. *Bacillus subtilis* was grown in 100-mL liquid medium overnight until the colony-forming units (CFU) per ml reached 10^7^–10^8^, of which 100 µl was taken and spread on LB agar plate. The solid agar medium was punctured using a tip, 6 mm in diameter, then, the 10 µl purified cecropin B was dropped into the holes. The plate was grown at 37 °C for 16 h, and transparent zone areas were measured to evaluate antimicrobial activity of the purified cecropin B peptides using ImageJ software.

## Results

### Constructions of 6xHisSUMO(3xGly)-cecropin B into pKSEC1 Expression Vector and Their Expression in *Escherichia coli*

The expression of SUMO-fused cecropin B was performed using pKSEC1 (Fig. 1A) which was developed in our lab previously [26]. The expression vector was intended to be usable in both *Escherichia coli* and plant chloroplasts. Since the plant chloroplast is a prokaryotic origin, its transcription and translation are similar to bacteria while there are some differences between them [41]. As shown in previous reports, chloroplast-derived *psbA* promoter/5′ UTR and 3′ UTR not only drives expression of transgenes strongly in chloroplasts but also in *Escherichia coli* [30, 31, 42]. In our previous study, our expression vector worked successfully in expressing SUMO-abaecin in *Escherichia coli* without affecting the host cell growth [26].

The vector has a SUMO tag, 96 amino-acid long in size, to increase solubility and prevent toxicity of AMPs to *Escherichia coli* hosts. Further, 6xHis tag is added to the N-terminus of SUMO for purification of the SUMO-fused AMPs and isolates AMPs after cleavage by SUMOase (Fig. 1A). Four different constructs were created to express cecropin B in a 6xHisSUMO fusion form (Fig. 1A). They were generated in a combination way using native (N) or codon-optimized (C) sequences: 6xHisSUMO-cecropin B (N-N), 6xHisSUMO-cecropin B (C-N) and 6xHisSUMO-cecropin B (C-C). The fourth one was the modified version, 6xHisSUMO3xGly-cecropin B (C-C), of 6xHisSUMO-cecropin B (C-C) in which three glycine amino acids are placed in between 6xHisSUMO and cecropin B. All the codon-optimized sequences were generated using the codon optimization algorithm [31].

To examine the effect of the codon optimized sequences on expression level of the fusion proteins, we performed western blot assays with total proteins extracted from transformed *Escherichia coli* cells with each of four constructs using anti-His antibody. Further, the extracted proteins were divided into soluble and insoluble fractions to investigate solubility of the fusion proteins using densitometry assay. The expressed fusion proteins were all detected at around 17 kDa which was a little bit higher than the theoretical molecular weight (15.9 kDa); however, the bands were rarely detected from insoluble fractions (Fig. 1B).

The codon-optimized sequences did not work better in enhancing translation than the construct with native sequences (Fig. 1B, C). The levels of expression of 6xHisSUMO (C)-cecropin B (N) and 6xHisSUMO (C)-cecropin B (C) failed to show the improvement in expression over the construct, 6xHisSUMO (N)-cecropin B (N). In contrast to 6xHisSUMO (C)-cecropin B (C), its modified version, 6xHisSUMO3xGly (C)-cecropin B (C), a little bit higher level of expression than 6xHisSUMO (N)-cecropin B (N).

From the results, the SUMO-fused cecropin B proteins are all soluble, but codon-optimization did not have a positive effect on the level of expression of the fusion proteins. However, the construct with a linker sequence (three glycines) at the interface between SUMO and cecropin B improved the expression level almost twice as much as its non-linker construct.

### Evaluation of Accessibility of SUMOase to Recognition Region Between SUMO and Cecropin B

**Fig. 2:**
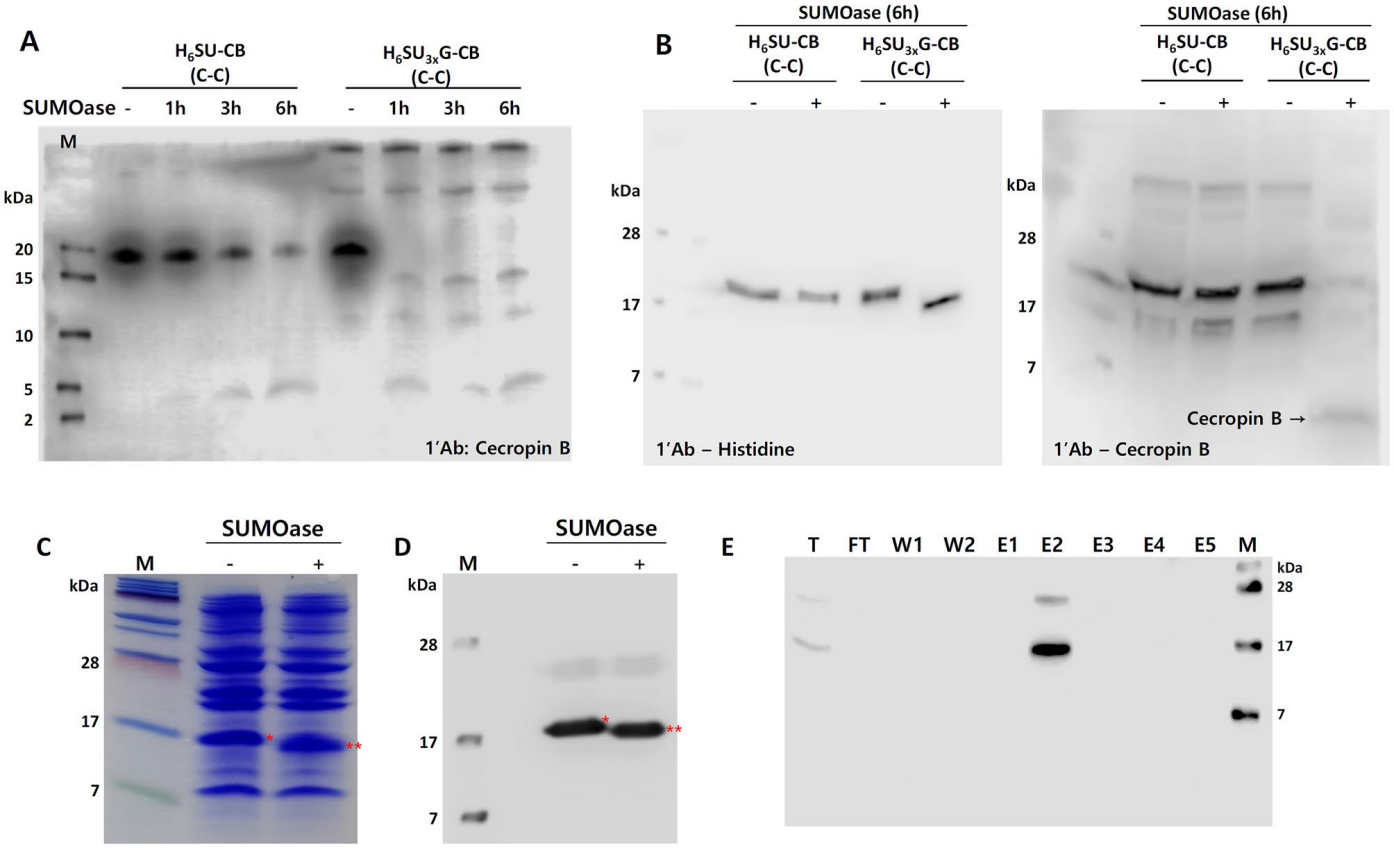
Western blot analysis for the comparison of expression level between 6xHisSUMO-cecropin B and 6xHisSUMO 3x Gly-cecropin B and purification of 6xHisSUMO 3x Gly-cecropin B fusion protein. **a** Cleavage assay of the fusion proteins, 6xHisSUMO-cecropin B and 6xHisSUMO3xGly-cecropin B, by SUMOase. **b** Western blot assay for the susceptibility effect of the addition of three glycine between SUMO and cecropin B by SUMOase. Purified fusion proteins from both H_6_SU-CB (6xHisSUMO-cecropin B) and H_6_SU_3x_G-CB (6xHisSUMO3xGly-cecropin B) were treated with SUMOase for 6 h, and 20 μg of proteins were loaded for each lane then immunoprobed using anti-histidine (left panel) and anti-cecropin B (right panel) antibody. **c** Coomassie staining assay and **d** western blot assay to investigate the recognizable cleavage by SUMOase with total proteins extracted from an *Escherichia coli* clone transformed with 6xHisSUMO3xGly-cecropin B construct. *6xHisSUMO3xGly-cecropin B; **cleaved 6xHisSUMO3xGly, no treatment of SUMOase; +, 5 h treatment of SUMOase. Each lane was loaded with 20 μg of total protein. **e** Western blot assay for the purified 6xHisSUMO3xGly-cecropin B from *Escherichia coli* using gravity Ni column. T, total protein; FT, flow-through; W, wash; E, elution sequentially separated by 1 mL

Next, the expressed fusion proteins were subject to SUMOase treatment to evaluate proper cleavage-off of SUMO by SUMOase. All four constructs of 6xHisSUMO-cecropin B have the same amino acid sequence except for the one which has additional three glycines at the interface. So, the fusion protein with no linker, 6xHisSUMO(C)-cecropin B(C), was compared with the one with the linker, 6xHisSUMO(C)3xGly-cecropin B(C). The purified fusion proteins were treated with SUMOase (1 unit per 20 μg) for up to 6 h at 30 °C. The fusion protein without linker showed the gradual release of cleaved cecropin B from SUMO tag over incubation time (Fig. 2A). In contrast, cleavage-off of the cecropin B from the fusion protein with a linker was much faster than that of the fusion protein with no linker so the release of cecropin B was accomplished within 1 h (Fig. 2A). To confirm that there was no non-specific activity of SUMOase on both SUMO and cecropin B, immunoblot assays were done with anti-histidine and anti-cecropin B antibodies against the fusion protein samples treated with SUMOase for 6 h (Fig. 2B). Anti-histidine antibody captured only the cleaved 6xHisSUMO located below 17.0 kDa after 6 h treatment from the fusion protein with the glycine linker (Fig. 2B, left panel), but, for the fusion protein with no linker, the same antibody just detected the 6xHisSUMO to which cecropin was still attached (Fig. 2B, left panel). The same protein samples were also immunoprobed by anti-cecropin B antibody. As shown in Fig. 2B (right panel), the released cecropin B from SUMO by SUMOase was clearly visible from the fusion protein with the linker, whereas the band for cecropin B was barely detected from the fusion protein without the linker. So, 6xHisSUMO3xG-cecropin B fusion protein was used for further study.

To evaluate the antimicrobial activity of cecropin B, the 6xHisSUMO3xGly-cecropin B expressed in *Escherichia coli* was purified and elution 2 fraction (E2, Fig. 2E) was chosen for further study because it showed the most enrichment of the fusion protein with a high purity. Before the purification, the selected *Escherichia coli* clone was verified whether the fusion protein was properly expressed in the *Escherichia coli* and the release of the cecropin B was reproducible when treated with SUMOase (Fig. 2C and D).

### Evaluation of Antibacterial Activity of Purified Cecropin B Against *Bacillus subtilis*

**Fig. 3:**
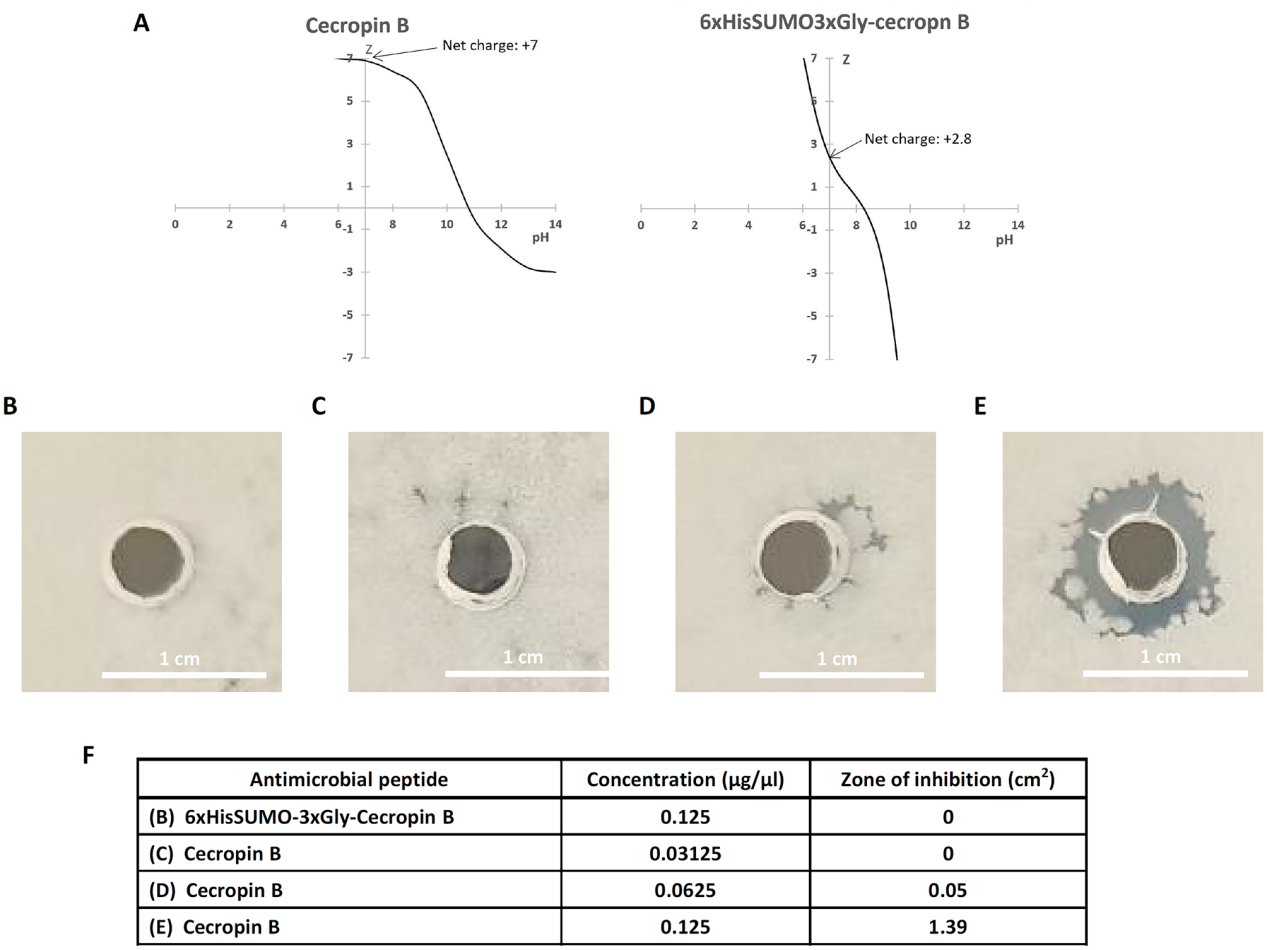
Shielding effect of positive charge of cecropin B by SUMO and evaluation of anti-bacterial activity of purified cecropin B. **a** Tritration curve of cecropin B (left panel) and 6xHisSUMO3xGly-cecropin B (right panel). *Z* represents net charge. Antibacterial activity of cecropin B was tested against *Bacillus subtilis*. Agar plates spread with 100 μL of *Bacillus subtilis* liquid culture grown overnight were punctured and dropped with 20 μL of purified cecropin B then incubated at 37 °C for 16 h. **b** 0.125 μg/μL 6xHis-SUMO-3xGly-cecropin B, **c** 0.03125 μg/μL cecropin B, **d** 0.0625 μg/μL cecropin B, and **e** 0.125 μg/μL cecropin B. **f** Table represents the inhibited zone areas of *Bacillus subtilis*, which were extrapolated using ImageJ software

Antimicrobial activity of AMPs comes from their hydrophobicity and high cationic charge. Expression of AMPs in *Escherichia coli* could be lethal to the host if the positive charges are not properly shielded. We analyzed off-set effect of the positive charge on the cecropin B by the fusion of SUMO tag. Cecropin B itself has +7 net positive charge (Fig. 3A, left panel) but the overall net charge becomes reduced to +2.8 once it is fused to 6xHisSUMO3xGly (Fig. 3A, right panel). This would be a possible explanation why the 6xHisSUMO3xGly-cecropin B fusion protein was not toxic to *Bacillus subtilis* as shown below.

In our previous study [26], we observed that cecropin B released from SUMO fusion protein with no linker showed almost no antibacterial activity against *Bacillus subtilis* in agar diffusion assay at a concentration of 0.125 μg/μL but showed the activity at a higher concentration, 0.25 μg/μL. However, in this study, we could observe the antibacterial activity at the concentration of 0.125 μg/μL (Fig. 3E) when we used the cecropin B released from 6xHisSUMO3xGly-cecropin B. Even at a concentration of 0.0625 μg/μL, the antimicrobial activity was detected although it was marginal but obvious (Fig. 3D), but no anti-bacterial activity was detected at a concentration of 0.03125 μg/μL (Fig. 3C). The increase of twofold concentration of cecropin B from 0.0625 μg/μL to 0.125 μg/μL increased zone of inhibition exponentially up to 28-fold, from 0.05 to 1.39 cm^2^, while the fusion protein, 6xHisSUMO3xGly-cecropin B, which was used as a control, showed no bacteriolytic activity when applied at the concentration of 0.125 μg/μL for 16 h (Fig. 3B).

Taken all together, 6xHisSUMO- or 6xHisSUMO3Gly-tagged cecropin B were all soluble when expressed in *Escherichia coli*, and those fusion proteins had no toxic effect to *Escherichia coli* hosts. The removal of SUMO tag was much more efficient when additional three glycines were added in between the SUMO and the cecropin B. In addition, the released cecropin B from the fusion protein with three-glycine linker showed higher antibacterial activity than the cecropin B released and purified from the non-linker containing fusion protein when compared both at the same concentration.

## Discussion 

As a countermeasure to emergence and the consequent increase of antibiotic-resistance pathogens, AMPs have been extensively explored because they have distinct features from conventional antibiotics. AMPs are very diverse in their structures and functions, and they have immunomodulatory activity as well [11]. Antibiotics also have a broad range of antibacterial spectrum but they have a narrow spectrum of bacteriolytic action modes, which is usually characterized with one defined, high affinity target, whereas AMPs are featured with multiple, low affinity targets. Therefore, resistance development by bacteria against AMPs is much more difficult compared to antibiotics [4].

As represented in recent studies with cecropins, AMPs can be applicable in various aspects, such as protecting crops [43], inhibiting tumor proliferation [44–46], modulating immune response [47, 48], as well as killing bacteria [12, 48–50]. As seen in some of these recent studies, therapeutic potency of cecropin was synergistically boosted by combination or hybrid with other molecules including abaecin [26], tetracycline [12], apoptin [45], magainin II [48], and lysozyme [50].

In our previous study, we confirmed that a cocktail treatment with abaecin and cecropin B showed the synergistic bacteriolytic effect against *Bacillus subtilis*. As an extension of our study, we attempted to increase the biomass of the recombinant cecropin B in *Escherichia coli* using our expression platform*.* The most notable finding was that the complete release of cecropin B from the SUMO tag was affected depending on the presence of linker sequence. The fusion protein with linker sequence showed complete and faster cleavage of SUMO upon treatment of SUMOase. In this study, we created an additional construct over the course of experiment, which has three glycine amino acids being placed in between the C-terminus of SUMO tag and N-terminus of cecropin B. Generally, linker sequences at the interface between two proteins relieve steric hindrance or increase solubility of a fusion protein [51]. In contrast to other proteolytic enzymes, SUMOase recognizes the tertiary structure of SUMO so the SUMOase does not leave any additional amino acids to a target protein [27]; otherwise, an undesired modification on target proteins can lead their inherent activity to be lost or less effective. This is one of the main reasons SUMO is largely chosen as a tag when expressing small size of peptides which could be more likely affected by an addition of even a single amino acid.

It could be inferred that the direct fusion of cecropin B to SUMO could cause some change in the tertiary structure of the SUMO, which makes full access of SUMOase to SUMO affected, resulting in the incomplete cleavage of SUMO from cecropin B despite the long hour treatment of SUMOase (Fig. 2A). However, the addition of a linker in between the SUMO and cecropin B eliminated the incompletion of the cleavage, which could be due to the removal of a steric hindrance caused by the direct addition of cecropin B to the C-terminus of SUMO.

Another finding in this study is that the released cecropin B from the SUMO with the linker showed improved anti-bacteriolytic activity over the one released from the fusion protein with no linker. In our previous study, anti-bacteriolytic activity of cecropin B was not observed at a concentration of 0.125 μg/μL [26], but showed some lethal activity in this study at the same concentration (Fig. 3). One possible explanation for the improved activity is that the removal of SUMO from the linker region did not happen precisely after 5th glycine (Fig. 1A). So, the cleavage happened after 4th or 3rd or 2nd glycine, which resulted in leaving cecropin B with an additional glycine or glycines on the N-terminus of cecropin B. The addition of glycine(s) could serve to enhance the antimicrobial potency of cecropin B, likely to increase the hydrophobicity of the cecropin B, which then help the cecropin B infiltrate into lipid layer to form pores. This explanation could sound quite conflicting to the description, specific cleavage activity by SUMOase, made above, but some specific amino acid sequence could clearly affect the SUMOase activity. As shown in a study, steric hindrance caused by insertion of bulky amino acid such as Tryptophan (W) near the cleavage site interferes with the approach of SUMOase to its action site, resulting in random cleavage within in a SUMO fusion protein, but SUMOase resumed its specific cleavage activity after the steric hindrance was relieved by addition of 1 M urea [34]. In this regard, it could be assumed that Trp located at 2nd place from N-terminus of cecropin B (Fig. 1A) serves as an obstacle to the access of SUMOase, which then reduces specific cleavage activity.

The toxicity of AMPs comes from their hydrophobicity and net positive charges, but at the same time, the highly cationic content of small peptides is prone to the proteolytic degradation in *Escherichia coli* [20]. These aspects need to be well controlled when manufacturing AMPs using *Escherichia coli* system. In this point, SUMO is well suited to tackle these unflattering issues. The net charge of SUMO (96 amino acids) and cecropin B (35 amino acids) is −4.8 and +7 (Fig. 3A, left panel), respectively, and the net charge of the fusion of the two proteins becomes +2.2 but, in this study, we added 6 histidines to the N-terminus of SUMO so the net charge of the fusion protein, 6xHisSUMO-cecropin B, is +2.8 (Fig. 3A, right panel). The offset effect of SUMO on the positive charge shows evident benefit for the expression of cecropin B. As seen in Fig. 3B, F, no toxicity was observed from the fusion protein, 6xHisSUMO-cecropin B, but the toxic effect of the cecropin B was restored when it was released from SUMO (Fig. 3D–F). In addition, we barely saw any degraded form of the fusion proteins in the course of purification (Fig. 2E), which indicates that the shielding of the positive charge of cecropin B by SUMO increased the stability of the AMP by protecting the cecropin B from enzymatic degradation.

We have explored the development of a new expression platform for manufacturing therapeutic AMPs using a prokaryotic-friendly vector system, equipped with SUMO and 6xHis as tags to enhance solubility, stability, detoxification, and purification of AMPs. In our consecutive studies with AMPs, we found that several points should be improved to move forward our system for practical use in mass production of AMPs. In our previous study, abaecin, fused to 6xHisSUMO, was not protected fully from proteolytic degradation in *Escherichia coli* [26], which seems to be due to the partial protection of abaecin by SUMO. One of two positively charged patches on abaecin was exposed to a protease, resulting in the 29-aa long abaecin derivative with C-terminal 5 amino acids being deleted. Engineering SUMO to be more negatively charged, so which allows for SUMO to protect AMPs with higher cationic charges, needs to be one of future studies. In addition, linker sequences that can relieve steric hindrance imposed on SUMO when attached to AMPs could be another study. We prefer using genetic engineering to relieve the steric hindrance, rather than using detergents such as Urea or DTT because the detergents should be completely removed before using AMPs for therapeutic purposes. Here, we use only three glycines as a linker to eliminate the steric hindrance. The linker sequence needs to be more studied to guarantee that the steric hindrance should not only completely eliminated but also any amino acids of linker should not be remained on target AMPs. We can assume that the complete removal of steric hindrance makes SUMO do its job better in protecting AMPs and also help provide SUMOase with full access to the cleavage site.

In the future, the genetic engineering of AMPs is required to develop more potent AMPs but this is a time- and labor-consuming job. Considering the current urgency to combat antibiotic-resistant bacteria, the cocktail therapy can be a more straightforward and realistic approach to battle with the resistance bacteria. As seen in a recent paper, an AMP teamed up with tetracycline shows enhanced killing effect on bacteria with sublethal dosage of each anti-bacterial agent. This synergistic therapeutic effect was also observed in our previous study that *Bacillus subtilis* was subject to death at a suboptimal dosage of each AMP in combinatorial treatment with abaecin and cecropin B [26]. Pores created in the bacterial membrane by cecropin B provide passage for abaecin to reach cytoplasm rapidly and target DnaK, a prokaryotic heat shock protein 70. Disastrous situations, which were generated by blockage of protein metabolism caused by the attack of abaecin to DnaK and by the leakage of metabolites and ions caused by pore-forming cecropin B, lead to the death of the bacteria with less use of each bacteriolytic agent than when each agent was applied alone. So various combinatorial treatment studies need to be pursued persistently to enrich our arsenal repository not to be subdued by antibiotic resistance bacteria.

To advance our expression platform further, it should be very useful taking advantage of the polycistronic expression nature of bacteria. In bacteria, most genes are clustered and many of them are expressed in a polycistronic mRNA form which is then translated to multiple proteins [52–54]. Once optimal combinatorial treatment against antibiotic resistance bacteria is empirically identified, those AMPs can be cloned in an operonic expression cassette and then multiple recombinant AMPs can be expressed simultaneously in *Escherichia coli*. This strategy will eventually reduce cost and resource when manufacturing customized therapeutic AMPs.

One disadvantage of using our expression platform is that AMPs having disulfide bridge(s) [55] are not suitable. Due to the reducing environment of cytoplasm in *Escherichia coli*, AMPs containing disulfide bonds should be sent to periplasmic space where the AMPs can be folded properly with a help of disulfide isomerase [56]. But this process requires high energy consumption which will eventually decrease the total yield of AMPs. SUMO has only one cysteine residue so there is no disulfide bond. When choosing an AMP to be expressed using our system, AMPs not requiring disulfide bond formation is favored.

From our continued studies with AMPs, we saw our expression system relevant for manufacturing AMPs in *Escherichia coli*. However, despite many advantages of using SUMO and affinity tags, several aspects, such as steric hindrance and incomplete protection of AMPs by SUMO, need to be improved in the next studies. So, this persistent effort should shorten the time for AMPs to reach clinical use for patients.

## Supplementary Information

Below is the link to the electronic supplementary material.

Supplementary file1 (PDF 1216 KB)

## Data Availability

All data generated or analyzed during this study are included in this published article and its supplementary information files.

## Abbreviations

AMP: Antimicrobial peptide
SUMO: Small ubiquitin-related modifier 1
