# Supplementary material for: Expression of Antimicrobial Peptide (AMP), Cecropin B, in a Fused Form to SUMO Tag With or Without Three-Glycine Linker in Escherichia coli and Evaluation of Bacteriolytic Activity of the Purified AMP
Source: Probiotics Antimicrob Proteins. 2021 May 20;13(6):1780–9. doi: 10.1007/s12602-021-09797-1 (PMC8578067; doi:10.1007/s12602-021-09797-1)
Supplement: Supplementary file 1 — Supplementary file1 (PDF 1216 KB) [file 12602_2021_9797_MOESM1_ESM.pdf]

1. Article title: Expression of antimicrobial peptide (AMP), cecropin B, in a fused form to SUMO tag with or without three-glycine linker in *Escherichia coli* and evaluation of bacteriolytic activity of the purified AMP.

2. Journal name: Probiotics and Antimicrobial Proteins

3. Author names: A Rom Park[1], Seon Woong Kim[1], Soon Young Kim[1*], Kwang-Chul Kwon[2*]

4. Affiliation:

[1]Department of Biological Sciences, Andong National University, Andong, Korea

[2]MicroSynbiotiX Ltd, 11011 N Torrey Pines Rd Ste. #135, La Jolla, CA, 92037, USA

4. Email address of the corresponding author: kwang-chul.kwon@microsynbiotix.com

Supplementary Fig. S1

A Sequence for 6xHis(N) SUMO(N)-*Cecropin B (N)*

CACCACCACCACCACCACTCTGACCAGGAGGCAAAACCTTCAACTGAGGACTTGGGGGATAAGAAGGAAGGTGAATATATTAAACTCAAAGT
CATTGGACAGGATAGCAGTGAGATTCACTTCAAAGTGAAAATGACAACACATCTCAAGAAACTCAAAGAATCATACTGTCAAAGACAGGGTGT
TCCAATGAATTCACTCAGGTTTCTCTTTGAGGGTCAGAGAATTGCTGATAATCATACTCCAAAAGAACTGGGAATGGAGGAAGAAGATGTGATT
GAAGTTTATCAGGAACAAACGGGGGGT*AAATGGAAAGTCTTCAAGAAAATTGAAAAAATGGGTCGCAACATTCGAAACGGTATTGTCAAGGC*
TGGACCAGCGATCGCGGTTTTAGGCGAAGCCAAAGCGCTA

B Sequence for 6xHis(C) SUMO(C)-*Cecropin B (N)*

CACCAtCAtCACCAtCACTCTGAtCAaGAaGCtAAACCTTCtACTGAaGAtcttGGtGATAAaAAaGAAGGTGAATATATTAAACTaAAAGTaATTGGA
CAaGATAGCAGTGAaATTCACTTCAAAGTaAAAATGACcACACATttgAAGAAAttaAAaGAATCcTACTGTCAAcgtCAaGGTGTTCCtATGAAcTCt
ttacgcTTcttgTTTGAGGGTCAacgtATcGCTGATAATCAcACTCCAAAgGAACTtGGAATGGAGGAAGAAGAcGTaATaGAAGTTTATCAGGAgCA
AACtGGcGGT*AAATGGAAAGTCTTCAAGAAAATTGAAAAAATGGGTCGCAACATTCGAAACGGTATTGTCAAGGCTGGACCAGCGATCGCGGT*
TTTAGGCGAAGCCAAAGCGCTA

C Sequence for 6xHis(C) SUMO(C)-*Cecropin B (C)*

CACCAtCAtCACCAtCACTCTGAtCAaGAaGCtAAACCTTCtACTGAaGAtcttGGtGATAAaAAaGAAGGTGAATATATTAAACTaAAAGTaATTGGA
CAaGATAGCAGTGAaATTCACTTCAAAGTaAAAATGACcACACATttgAAGAAAttaAAaGAATCcTACTGTCAAcgtCAaGGTGTTCCtATGAAcTCt
ttacgcTTcttgTTTGAGGGTCAacgtATcGCTGATAATCAcACTCCAAAgGAACTtGGAATGGAGGAAGAAGAcGTaATaGAAGTTTATCAGGAgCA
AACtGGcGGT*AAATGGAAgGTaTTCAAGAAAATTGAgAAAATGGGTCGCAACATcCGtAAtGGTATTGTaAAGGCTGGACCtGCtATCGCtGTTTT*
AGGCGAAGCtAAAGCaCTA

D Sequence for 6xHis(C) SUMO(C)-3xGly(C)-*Cecropin B (C)*

CACCAtCAtCACCAtCACTCTGAtCAaGAaGCtAAACCTTCtACTGAaGAtcttGGtGATAAaAAaGAAGGTGAATATATTAAACTaAAAGTaATTGGA
CAaGATAGCAGTGAaATTCACTTCAAAGTaAAAATGACcACACATttgAAGAAAttaAAaGAATCcTACTGTCAAcgtCAaGGTGTTCCtATGAAcTCt
ttacgcTTcttgTTTGAGGGTCAacgtATcGCTGATAATCAcACTCCAAAgGAACTtGGAATGGAGGAAGAAGAcGTaATaGAAGTTTATCAGGAgCA
AACtGGcGGTGGaGGaGGc*AAATGGAAgGTaTTCAAGAAAATTGAgAAAATGGGTCGCAACATcCGtAAtGGTATTGTaAAGGCTGGtCCtGCtA*
TCGCtGTTTTAGGtGAAGCtAAAGCaCTA

Supplementary Fig. S1 Sequences of four different 6xHisSUMO(3xGly)-cecropin B constructs. 6xHis and 3xGly sequences are underlined, SUMO sequences are represented with standard font and the sequences of cecropin B are italicized. N, native sequence; C, codon-optimized sequence.
